# ZNF224, Krüppel like zinc finger protein, induces cell growth and apoptosis-resistance by down-regulation of p21 and p53 via miR-663a

**DOI:** 10.18632/oncotarget.8870

**Published:** 2016-04-20

**Authors:** Jin Gu Cho, Seho Park, Chae Hyun Lim, Hong Sook Kim, Seung Yong Song, Tae-Young Roh, Jong-Hyuk Sung, Wonhee Suh, Seok-Jin Ham, Key-Hwan Lim, Sang Gyu Park

**Affiliations:** ^1^ Department of Biomedical Science, CHA University, Sungnam-si, Gyunggi-do, Korea; ^2^ Laboratory for Tracing of Gene Function, Department of Pharmacy, College of Pharmacy, Ajou University, Suwon, Gyunggi-do, Korea; ^3^ Department of Surgery, Yonsei University College of Medicine, Seoul, Korea; ^4^ Division of Integrative Biosciences & Biotechnology, Pohang University of Science & Technology (POSTECH), Pohang, Gyeongbuk, Korea; ^5^ Department of Plastic and Reconstructive Surgery, Yonsei University College of Medicine, Seoul, Korea; ^6^ Department of Pharmacy, College of Pharmacy, Yonsei University, Incheon, Korea; ^7^ Department of Pharmacy, College of Pharmacy, Chung-Ang University, Seoul, Korea

**Keywords:** ZNF224, miR-663a, p53, p21, apoptosis

## Abstract

ZNF224 is a Krüppel-associated box-containing zinc-finger protein which represses gene transcription by interacting with various co-repressors. However, its consensus DNA sequences and target genes are not fully identified. In this study, we identified and characterized consensus DNA sequences containing 5′-CAGC-3′; recognized by ZNF224 through ChIP-sequencing, which further confirmed by ELISA, SPR, qPCR, and luciferase activity assay. ZNF224 increased miR-663a transcription by binding to miR-663a promoter, which in turn binds to 3′; UTR of p53 and p21 to decrease their expression. miR-663a antagonist abolished ZNF224-mediated suppression of p21 and p53, resulting in the enhanced apoptosis by CPT. The analyses using human breast ductal carcinoma tissues exhibited that the expression of ZNF224 and miR-663a was increased in cancer compared to non-cancer region. Consequently, ZNF224 increases cell survival and decreases apoptosis by decreasing the expression of p53 and p21 via miR-663a as a transcriptional activator. Taken together, we identified and characterized DNA binding element of ZNF224, and its target genes, miR-663a, which provides a novel insight in the down-regulation of p21 and p53 via miR-663a by ZNF224 in breast cancer.

## INTRODUCTION

Transcription factor (TF) represses or activates transcription of target genes by binding to its DNA binding elements during a variety of cellular processes. Krüppel-like associated box-zinc finger proteins (KRAB-ZFPs), is a member of DNA binding protein family [1]. KRAB-ZFPs have conserved KRAB domain at N-terminal region and a C_2_H_2_-type zinc-finger motif at C-terminal region [2]. KRAB-ZFPs have one or more KRAB A boxes, and/or a KRAB B box, or a SCAN-domain, a leucine-rich region that is found in a small subset of KRAB-ZFPs [3–4]. Recently, it has been known that KRAB domain of ZFP binds to corepressor proteins and/or TFs via protein-protein interactions, resulting in transcriptional repression of target genes [5–6]. KRAB-ZFPs forms complex with KRAB-associated protein 1 (KAP1), one of corepressor as well as a mediator of chromatin remodeling complex, then binds to its corresponding DNA consensus sequence to silence gene expression [7–9].

ZNF224 is a transcription factor composed of KRAB A domain, KRAB B domain at N-terminal region, and 19 tandemly repeated C_2_H_2_-type zinc finger motifs [10]. ZNF255, an alternative splice variant of ZNF224, lacks the KRAB domain, but contains 19 zinc-finger domains [11]. There are some differences in the localization. ZNF224 is homogenously distributed in the nucleus, whereas ZNF255 localized in subnuclear structures in association with nucleoli, and also in the cytoplasm [12]. The nuclear interaction of ZNF224 with Wilms’ tumor suppressor gene (WT1) increases the expression of WT1 target genes, whereas ZNF255 mainly participates in RNA maturation and processing, together with WT1 [13].

ZNF224 functions as a transcriptional repressor by forming a complex with KAP1 and a type II protein arginine methyltransferase (PRMT5), leading to transcriptional repression of aldolase A gene [10, 14–16]. In addition, ZNF224 represses transcription of mitochondrial citrate carrier gene [17]. Interestingly, ZNF224 acts as a co-activator of WT1 in the regulation of proapoptotic and antiapoptotic genes, including Bax, Bak, Vitamin D receptor (VDR), bag3, and A1/Bfl1 [5]. Moreover, DEP domain containing 1(DEPDC1) suppressed A20 transcription by ZNF224. However, upon disruption of ZNF224/DEPDC1 complex, the A20 mRNA expression is increased [18]. These results suggest that ZNF224 functions as an oncoprotein or tumor suppressor in interacting protein dependent manner or in cancer type dependent manner.

In this study, we first determined whether ZNF224 could function as an oncogenic protein. In addition, we tried to identify target genes and DNA consensus sequence recognized by ZNF224 in MCF-7 cells. We performed ChIP sequencing and then, identified and characterized DNA consensus sequence. Interestingly, we found that this consensus sequence recognized by ZNF224 is present within - 500 bp of miR-663a promoter, and examined whether ZNF224 increases or decreases expression of miR-663a. Furthermore, we assessed the expression of ZNF224 and miR-663a using human breast cancer tissues.

## RESULTS

### ZNF224 increases cell growth and drug resistance in MCF-7

**Figure 1:**
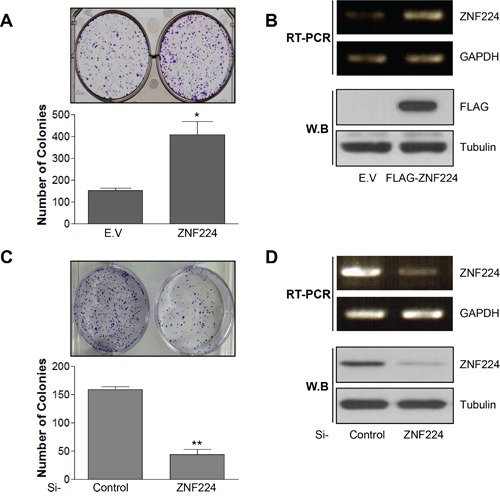
ZNF224 increases colony forming ability of MCF-7 cells **A.** MCF-7 cells were transfected with empty vector (E.V) or FLAG-ZNF224 (2 μg) and subjected to colony forming assay for 14 days (*, vs. E.V). **B.** The over-expression of FLAG-ZNF224 was confirmed by RT-PCR and immunoblot. **C.** MCF-7 cells were transfected with control or ZNF224 targeting si-RNA (20 nM), and colony forming ability was examined (**, vs. si-control). **D.** The knock-down of ZNF224 was examined by RT-PCR and immunoblot. GAPDH and tubulin were used as loading control. Data represent the mean ± SEM of three independent experiments. *P < 0.01, and **P < 0.01.

**Figure 2:**
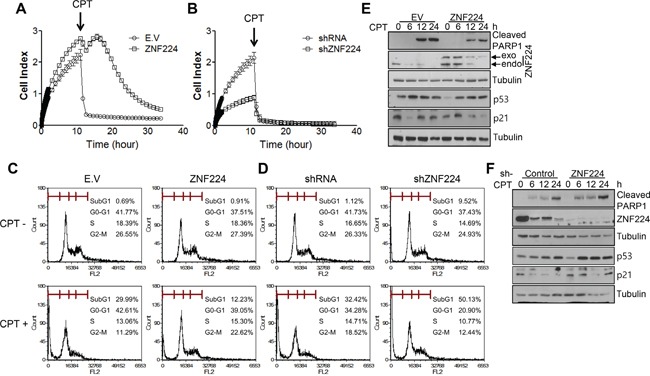
ZNF224 increases cell growth and apoptosis resistance **A** and **B.** ZNF224 over-expression cell line or ZNF224 knock-down cell line was seeded into iCelligence chamber and cultured for 10 h. Then CPT (0.1 μM) was treated, and cell growth was analyzed. Data are from at least three independent experiments (n=3). **C** and **D.** ZNF224 over-expression cell line or ZNF224 knock-down cell line was treated with CPT (0.1 μM) for 24 h, and subG1 population was analyzed as described in the Methods section. Data are from at least three independent experiments (n=3). **E** and **F.** ZNF224 over-expression cell line or ZNF224 knock-down cell line was treated with CPT (0.1 μM) in a time-dependent manner. Protein extracts were subjected to SDS-PAGE to analyze PARP1 cleavage. Tubulin was used as a loading control.

To determine whether ZNF224 functions as an oncogene or tumor suppressor, ZNF224 was transfected into MCF-7 cells and subjected to colony forming assay. ZNF224 over-expression increased colony forming ability about 3 folds, which was decreased about 4 folds upon si-RNA knock-down of ZNF224 (Figure 1A–1D). These results suggest that ZNF224 could function as an oncogene. Since over-expression of oncogene induces resistance to apoptosis [19], we generated ZNF224 over-expressing cell line and ZNF224 knock-down cell line, respectively, as described in the Methods section, and examined cell survival against CPT, a DNA damaging agent, using iCelligence, and apoptosis using FACS. ZNF224 over-expression showed enhanced proliferation compared to E.V control (Figure 2A), whereas the ZNF224 knock-down showed the reduced cell proliferation compared to control shRNA (Figure 2B). ZNF224 over-expression delayed apoptosis compared to E.V control upon CPT treatment (Figure 2A), which increased apoptosis upon knock-down of ZNF224 using shRNA (Figure 2B). In addition, apoptosis analysis using FACS showed that subG1 population induced by CTP in ZNF224 over-expression cell line was lower than E.V control (Figure 2C), whereas subG1 population of ZNF224 knock-down cell line was higher than control shRNA (Figure 2D). PARP1 cleavage assay further clearly showed that ZNF224 over-expression cell line is more resistant to CPT-induced apoptosis compared to E.V control, and vice versa, upon shRNA knock-down of ZNF224 (Figure 2E and 2F).

### Determination of DNA consensus sequences and its target gene recognized by ZNF224

**Figure 3:**
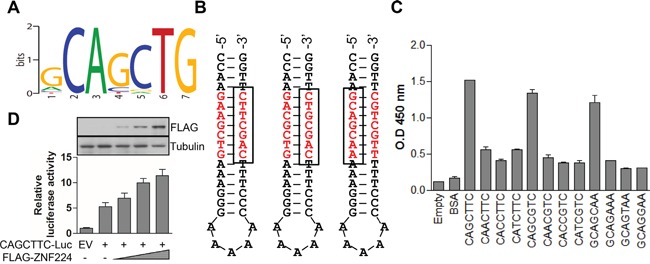
ZNF224 DNA binding motif characterization **A.** DNA motif within ZNF224 enriched region from ChIP sequencing. **B.** Hair-pin loop structure containing 5′-CAGC-3′ sequence. **C.** Biotinylated oligo DNAs (5 μM) were fixed to streptavidin coated 96 well plates and purified FLAG-ZNF224 (50 ng) was loaded to each well. The bound FLAG-ZNF224 was detected with anti-FLAG antibody, and developed as described in the Methods section. Data are from at least three independent experiments (n=3). **D.** pGL3 vector containing (200 ng) 5′-CAGCTTC-3′ sequence with empty vector (E.V) or FLAG-ZNF224 (50, 100, and 200 ng) was transfected to MCF-7 cells for 24 h, and luciferase activity assay was performed. Luciferase activity was normalized to *Renilla* (100 ng) luciferase activity. The expression of FLAG-ZNF224 was detected with anti-FLAG antibody (inset). Tubulin was used as a loading control. Data are from at least three independent experiments (n=3).

As described above, it is postulated that ZNF224 can function as an oncogene in MCF-7 cells. However, its DNA consensus sequence or target genes are not fully unveiled. Thus we tried to identify DNA consensus sequence and its target genes of ZNF224 through ChIP sequencing. Using the HOMER, we identified 10,185 peaks of ZNF224 in HEK293, and there was a tendency for ZNF224 enriched regions within 1 kb of the 5′ gene boundary to cluster around transcriptional start site (TSS) (Supplementary Figure 1A). The genes containing ZNF224 enriched regions near (<1 kb) are listed in Supplementary Table 1. In addition, as shown in Supplementary Figure 1B, the ZNF224 signals at promoters including 1 kb upstream and downstream of the TSS occupy about 12% of total peaks in HEK293. To determine the DNA consensus sequence recognized by ZNF224, 13,561 peaks validated by the MACS program were analyzed using HOMER, and further processed using Structural Time series Analyser, Modeller and Predictor (STAMP). We found a putative motif containing the sequence 5′-CAGC-3′ as shown in Figure 3A. We then synthesized a variety of 5′-biotinylated hairpin duplex oligo DNA (Figure 3B), and purified FLAG-ZNF224 from HEK293 cells. To confirm the binding of ZNF224, biotinylated oligo DNA was fixed to streptavidin coated plate, and then purified FLAG-ZNF224 was added to each well. Then the bound FLAG-ZNF224 was detected with anti-FLAG antibody. As shown in Figure 3C, the sequences containing 5′-CAGC-3′ showed significantly higher binding affinity compared to control or mutant oligo DNAs. In addition, to further confirm the binding of ZNF224 to oligo DNA, we performed the SPR assay. Quantitation of the binding affinity of ZNF224 to oligo DNA containing 5′-CAGC-3′ showed that the ZNF224 bound to CAGCTTC (K_D_= 1.25±0.06×10^−6^ M), CAGCGTC (K_D_=2.0±0.1×10^−6^ M), and GCAGCAA (K_D_=0.42±0.03×10^−6^ M), whereas oligo DNA containing mutant sequence didn't show any binding affinity (data not shown). To determine whether the oligo DNA sequence containing 5′-CAGC-3′ can function as promoter, we constructed luciferase vector containing 5′-CAGCGTC-3′ sequence in the promoter as a representative. As shown in Figure 3D, ZNF224 increased luciferase activity in a dose-dependent manner, confirming that DNA sequence containing 5′-CAGC-3′ functions as DNA consensus sequence recognized by ZNF224 in the promoter. Then we searched for DNA sequence containing 5′-CAGC-3′ among promoter enriched tags of ChIP sequencing and summarized top 10 candidates (Supplementary Figure 2A), and 7 of 10 candidate genes shows agreement with top 10 ChIP tags (Supplementary Figure 2B).

### ZNF224 decreases expression of p53 and p21 via miR-663a

**Figure 4:**
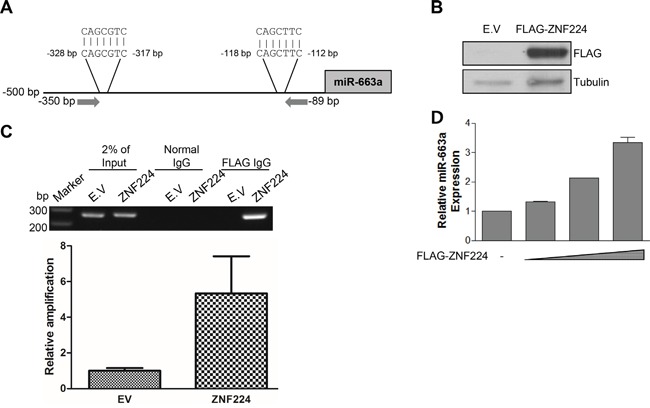
ZNF224 binds to miR-663a promoter **A.** miR-663a promoter sequence analysis. Two regions containing 5′-CAGC-3′ sequence were found within - 500 bp. Arrow indicates primer binding sites for PCR. **B** and **C.** FLAG-ZNF224 was transfected to HEK293 cells, and ChIP assay using normal or FLAG IgG was carried out. PCR was performed using miR-663a promoter primers between -350 bp and -89 bp. In addition, ChIPed DNA was analyzed with qPCR. Data are from at least three independent experiments (n=3). **D.** FLAG-ZNF224 (0.5 1, and 2 μg) was transfected to MCF-7 cells in a dose-dependent manner for 24 h, and miR-663a transcript level was examined by qRT-PCR. miR-663a transcript level was normalized to U6 RNA. Data are from at least three independent experiments (n=3).

It has been known that miR-663a was highly expressed in lung and prostate cancer [20–21]. The increased expression level of miR-663a is associated with poor prognosis [20], and the down-regulation of miR-663a sensitized chemoresistant breast cancer cells to cyclophosphamide and docetaxel [22]. In addition, miR-663a regulates cancer cell proliferation by targeting TGFβ 1 and p21 [21, 23]. Furthermore, the expression of miR-663a was increased in nasopharyngeal carcinoma [23]. However, in other cancers, including gastric cancer, leukemia, non-small cell lung cancer, pancreatic cancer, multiple myeloma, and glioblastoma, miR-663a functions as a tumor suppressor [24–31]. Of note, miR-663a was shown to have two putative DNA consensus sequences recognized by ZNF224 in -500 bp region as shown in Figure 4A. To further confirm the binding of ZNF224 to the promoter of miR-663a, we transfected FLAG-ZNF224, performed ChIP assay, and PCR. As shown in Figure 4B and 4C, anti-FLAG antibody precipitated the promoter DNA of miR-663a, but not mock antibody. In addition, qRT-PCR analysis in MCF-7 showed that ZNF224 increased transcription level of miR-663a in a dose-dependent manner (Figure 4D). These results suggest that ZNF224 increases transcription of miR-663a by binding to the promoter containing 5′-CAGC-3′ sequence.

**Figure 5:**
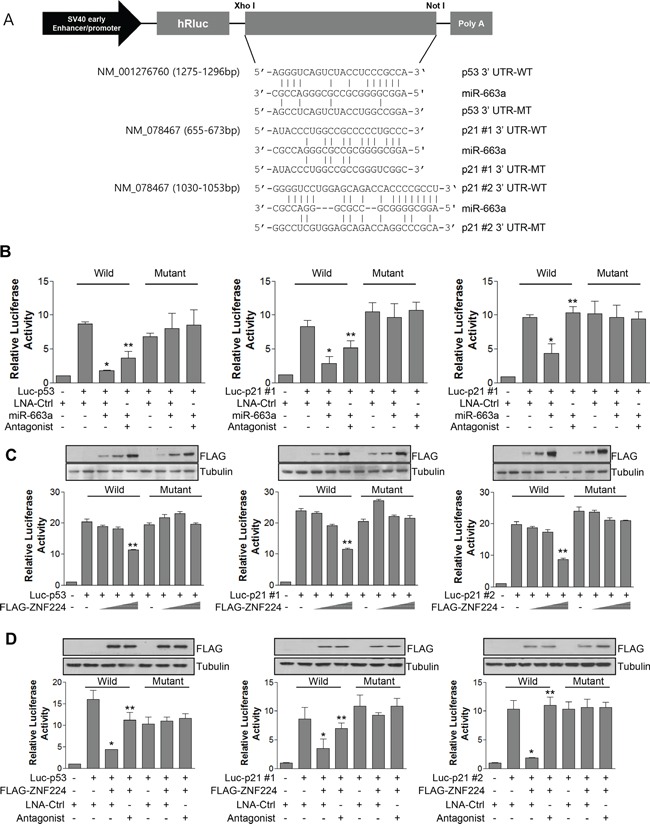
ZNF224 regulates promoter activity of p21 and p53 via miR-663a **A.** the putative miR-663a binding sequences for the p53 and p21 3′ UTRs. Human p53 and p21 3′; UTR fragments containing either the wild type (WT) or mutant (MT) miR-663a binding sequences were cloned downstream of the luciferase reporter gene in the psiCHECK vector. **B.** MCF-7 cells were co-transfected with psiCHECK vector (200 ng) and *Renilla* vector (100 ng) in the presence or absence of miR-663a (20 nM), and in the presence or absence of miR-663a antagonist (40 nM). Luciferase activity was normalized to *Renilla* luciferase activity. Data represent the mean ± SEM of three independent experiments (*, vs. luc-p53 or luc-p21 #1 or luc-p21 #2/LNA-control (ctrl); **, vs. luc-p53 or luc-p21 #1 or luc-p21 #2/LNA-ctrl/miR-663a). **C.** MCF-7 cells were co-transfected with psiCHECK vector (200 ng) and *Renilla* vector (100 ng) in the presence or absence of FLAG-ZNF224. Luciferase activity was normalized to *Renilla* luciferase activity. The expression of FLAG-ZNF224 was confirmed using anti-FLAG antibody (inset). Tubulin was used as a loading control. Data represent the mean ± SEM of three independent experiments (**, vs. luc-p53 or luc-p21 #1 or luc-p21 #2/FLAG-ZNF224 (−)). **D.** MCF-7 cells were co-transfected with psiCHECK vector (200 ng) and *Renilla* vector (200 ng) in the presence or absence of FLAG-ZNF224, and in the presence or absence of miR-663a antagonist (40 nM). Luciferase activity was normalized to *Renilla* luciferase activity. The expression of FLAG-ZNF224 was confirmed using anti-FLAG antibody (inset). Tubulin was used as a loading control. Data represent the mean ± SEM of three independent experiments (*, vs. luc-p53 or luc-p21 #1 or luc-p21 #2/LNA-ctrl; **, vs. luc-p53 or luc-p21 #1 or luc-p21 #2/FLAG-ZNF224/LNA-ctrl). *P < 0.001, **P < 0.01.

miR-663a binds to the 3′ UTR of p21 to down-regulate its expression in nasopharyngeal carcinoma [23], and we also found that p53 contains miR-663a binding sequence in 3′ UTR (Figure 5A). Therefore, we constructed luciferase reporter vectors and examined whether the miR-663a could down-regulate the expressions of p53 and p21. As shown in Figure 5B, miR-663a decreased luciferase activity of p53-3′ UTR-wild type, and p21-3′ UTR-wild type, but not of p53-3′ UTR-mutant, and p21-3′ UTR-mutant, agreement with previous report [23]. In addition, miR-663a antagonist abrogated the decrease of luciferase activity by miR-663a. Furthermore, ZNF224 decreased luciferase activity of p53-3′ UTR-wild type, and p21-3′ UTR-wild type in a dose-dependent manner, but not of p53-3′ UTR-mutant, and p21-3′ UTR-mutant (Figure 5C). To confirm whether the decreased luciferase activity of p53-3′ UTR and p21-3′ UTR is mediated by miR-663a via ZNF224, we examined luciferase activity after transfection of ZNF224 and miR-663a antagonist. The over-expression of ZNF224 significantly decreased luciferase activity of p53-3′ UTR-wild type, and p21-3′ UTR-wild type, but not of p53-3′ UTR-mutant, and p21-3′ UTR-mutant (Figure 5D). In addition, miR-663a antagonist significantly abolished the decrease of luciferase activity by ZNF224 (Figure 5D), suggesting that ZNF224 can down-regulate the expression of p53 and p21 by increasing the transcription of miR-663a.

**Figure 6:**
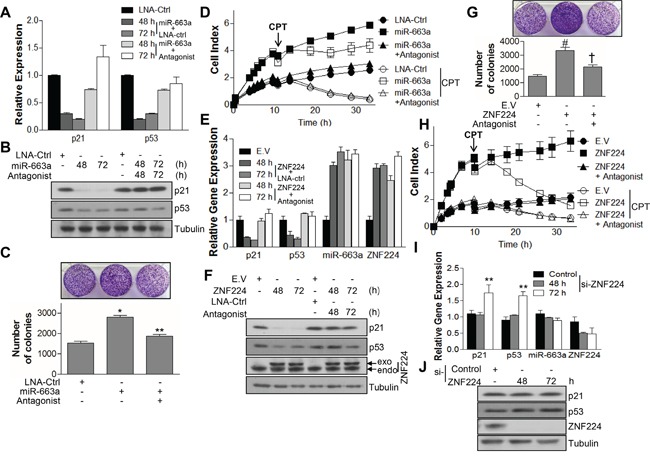
ZNF224 controls cell growth by regulating expressions of p21 and p53 via miR-663a **A** and **B.** MCF-7 cells were transfected with LNA-ctrl or miR-663a (20 nM) in the presence or absence of miR-663a antagonist (40 nM) as indicated. The expression of p21 and p53 was examined by qRT-PCR and immunoblot, respectively. GAPDH and tubulin were used as loading control in qRT-PCR and immunoblot, respectively. Data are from at least three independent experiments (n=3). **C** and **D**. In addition, colony forming ability was analyzed (*, vs. LNA-ctrl; **, vs. miR-663a) and subjected to iCelligence for the growth analysis in the presence or absence of CPT (0.1 μM). **E** and **F.** MCF-7 cells were transfected with FLAG-ZNF224 (2 μg) in the presence or absence of miR-663a antagonist (40 nM). The expression of p21, p53, miR-663a, and ZNF224 was examined by qRT-PCR and immunoblot, respectively. GAPDH and U6 RNA were used as loading control for mRNA and microRNA, respectively, in qRT-PCR, and tubulin was used as a loading control for immunoblot. Data are from at least three independent experiments (n=3). **G** and **H**. In addition, colony forming ability was analyzed (#, vs. EV; †, vs. ZNF224) and subjected to iCelligence for the growth analysis in the presence or absence of CPT (0.1 μM). **I** and **J**. MCF-7 cells were transfected with control or ZNF224 si-RNA (20 nM), and the expression of p21, p53, miR-663a, and ZNF224 was examined by qRT-PCR and immunoblot, respectively (**, vs. control si-RNA). GAPDH and U6 RNA were used as loading control for mRNA and microRNA, respectively, in qRT-PCR, and tubulin was used as a loading control for immunoblot. Data represent the mean ± SEM of three independent experiments. *P < 0.01, **P < 0.01, # *P* < 0.01, and † *P* < 0.01.

Next, we examined whether ZNF224 could decrease the transcript and protein level of p53 and p21 via miR-663a. miR-663a transfection efficiently decreased protein level as well as transcript level of p21 and p53 (Figure 6A and 6B). In addition, miR-663a increased colony forming ability of MCF-7 cells (Figure 6C), whereas miR-663a antagonist abrogated the decreased expression of p21 and p53, and increased colony formation induced by miR-663a (Figure 6A–6C). Since the decreased expression level of p21 and p53 induces apoptosis resistance [32], cell growth and apoptosis were analyzed after treatment of CPT. miR-663a transfection increased cell growth of MCF-7 cells and induced resistance of apoptosis by CPT, and vice versa, upon transfection of miR-663a antagonist (Figure 6D). In addition, transfection with ZNF224 decreased not only mRNA levels of p21 and p53 but also their protein levels, and increased transcript level of miR-663a, whereas miR-663a antagonist abrogated the effect of ZNF224, suggesting that the decreased expression of p21 and p53 by ZNF224 was mediated by miR-663a (Figure 6E and 6F). In addition, the increased colony forming ability and cell growth induced by ZNF224 over-expression was abolished by transfection of miR-663a antagonist (Figure 6G and 6H). Moreover, miR-663a antagonist abrogated the enhanced cell growth induced by ZNF224 over-expression, leading to sensitization of apoptosis resistance (Figure 6H). Si-RNA transfection of ZNF224 increased transcript levels of p21 and p53, but not miR-663a transcript level (Figure 6I). Western blot analysis showed that knock-down of ZNF224 using si-RNA increased the expression level of p53, but not p21 (Figure 6J).

**Figure 7:**
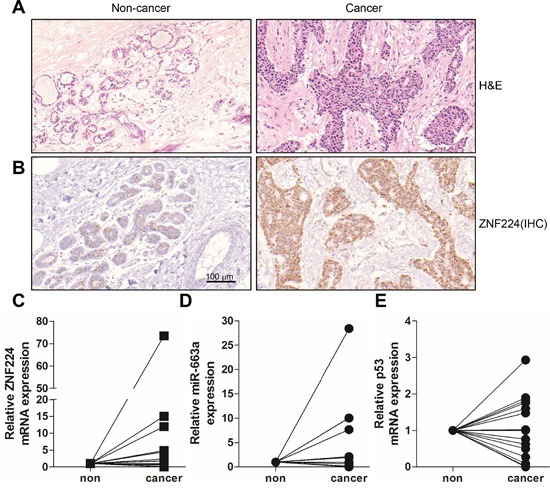
Expression analyses in human breast cancer **A** and **B.** Tissues of breast cancer patients were divided into non cancer and cancer region, and H&E staining and immunohistochemistry using anti-ZNF224 antibody was carried out. **C-E.** Total RNAs were isolated from cancer and non-cancer regions of breast cancer patients, and the expression of ZNF224, miR-663a, and p53 was examined by qRT-PCR (n=14-18). GAPDH and U6 were used as loading control in ZNF224 and p53, and miR-663a, respectively.

Next, we examined the transcript levels of ZNF224, miR-663a, p21, and p53 in ductal carcinoma of breast cancer patients. H&E and immunohistochemical staining showed that ZNF224 expression level was increased in cancer region compared to non-cancer region (15 of 18 cases) (Figure 7A and 7B), whereas ZNF224 mRNA expression was increased only in 8 cases of 18 cases (Figure 7C). In addition, miR-663a transcript level was increased in 7 of 14 cases, and p53 mRNA level was decreased in 8 cases of 16 cases (Figure 7D and 7E), whereas p21 mRNA was not detected (data not shown). Among the tested tissues, 3 cases (16.6%) showed that the increase of ZNF224 transcript level is correlated with increased miR-663a RNA (r_s_ = −0.4862, p=0.0389) and decreased p53 mRNA (r_s_ = −0.3446, p=0.046).

## DISCUSSION

Although ZNF224, one of KRAB-ZFPs, has been known as a transcriptional repressor of aldolase A, mitochondrial citrate carrier, and A20 [10, 14, 17–18], ZNF224 acts as co-activator of WT1 to increase the gene expression, including Bax, Bak, Vitamin D receptor (VDR), bag3, and A1/Bfl1 [33]. DNA consensus sequences of KRAB-ZFPs have been known [34–37], however, their consensus sequences don't show any similarity between KRAB-ZFPs. Therefore, to unveil the precise mechanism of ZNF224 as an oncogene or tumor suppressor gene, and because it is not known even whether ZNF224 binds to which sequences, we tried to identify target genes and DNA consensus sequence recognized by ZNF224. We found that DNA consensus sequences containing 5′;-CAGC-3′; is critical to the binding of ZNF224, which was further characterized by ELISA, SPR and luciferase activity assay. This DNA consensus sequence is different in length from other consensus sequences that show over 10 bases.

miR-663a, identified as a target gene of ZNF224, exhibits a differential function in different cancer types, as a tumor suppressor or as an oncogene [20–29, 31]. In this study, we identified miR-663a as an oncogene in breast cancer by directly down-regulating p21 and p53. Why miR-663a shows differential activity in a variety of cancer may be due that the mRNA copy number and genes of miR-663a target is different in cancer. In addition, it might be possible that the order of target genes could be determined by base pairing affinity with miR-663a because base paring between mircro-RNA and its target gene varies from 4 base pairs to 9 base pairs.

The over-expression of ZNF224 directly increased transcription level of miR-663a. However, knock-down of ZNF224 using si-RNA didn't decrease miR-663a transcript level, and increased p21 and p53 mRNA levels, whereas p53 expression is increased (Figure 6I and 6J); this is because the reduced expression of ZNF224, a transcription factor, might indirectly increase p53 expression by affecting transcriptional network. It has been known that miR-125b, miR-130b, and miR-155 promote tumor growth by decreasing tumor protein 53-induced nuclear protein 1 (TP53INP1) that increases p53 expression [38–41]. We found that the knock-down of ZNF224 using si-RNA decreased expression of miR-125b, miR-130b, and miR-155 in microarray (Supplementary Figure 3), and increased TP53INP1α expression in immunoblot. In addition, the over-expression of FLAG-ZNF224 decreased expression of TP53INP1α, but not TP53INP1β (Supplementary Figure 4A and 4B), which shows a possibility that ZNF224 could decrease p53 expression by TP53INP1α via miR-125b, miR-130b, and miR-155 as well. In addition, DNA consensus sequences containing 5′-CAGC-3′ was found in the promoter of p21 and p53, and PCR after ChIP using anti-FLAG antibody showed that ZNF224 could bind to the promoter of p21 and p53, suggesting that ZNF224 might function as a transcriptional repressor of p21 and p53 (Supplementary Figure 5A and 5B). Furthermore, CPT treatment induced degradation of ZNF224 in a time dependent manner, and after then, the mRNA levels of p21 and p53 were increased (Supplementary Figure 6A and 6B), which further suggests a possibility that ZNF224 might function as a transcriptional repressor of p21 and p53.

Interestingly, CPT treatment decreased expression level of ZNF224 in 6 h, and scarcely detectable after 24 h (Figure 2E and 2F), which suggest that DNA damage signal induces transcriptional repression or protein turn-over of ZNF224. However, qRT-PCR of ZNF224 after CPT treatment did not induce any change of ZNF224 mRNA level (data not shown), which suggests a possibility that DNA damage signal may induce degradation of ZNF224 by post-translational modification such as ubiquitination. It is well known that KRAB domain and it's neighboring amino acid sequences are critical to ubiquitination of KRAB-ZFPs [42–43]. Interestingly, KAP1 binds to KRAB domain of KRAB-ZFPs to function as an ubiquitin E3 ligase [43–44]. In another study, it has been reported that the expression of KAP1 and KRAB-ZFPs was increased in breast carcinoma, and sumoylated-KAP1 protects the KRAB-ZFPs from proteasomal degradation, which promotes proliferation and metastatic progression of breast cancer cells [45]. Therefore, whether DNA damage signal induces degradation of ZNF224 in a KAP1 dependent manner or by another mechanism needs further investigation.

In addition, immunohistochemical staining showed that ZNF224 expression level was increased in most cancer region (15 of 18 cases) (Figure 7A and 7B), whereas ZNF224 mRNA expression was increased only in 8 cases of 18 (Figure 7C). Addison *et al* reported that mRNA expression level of ZNF224 is decreased in cancer compared to normal area [45]. This result also suggests a possibility that ZNF224 could be stabilized by posttranslational modification, not transcription level, in cancer. Therefore, the stable over-expression of ZNF224 in breast cancer might induce apoptosis resistance, leading to cancer progression by down-regulating the expression of p21 and p53 via miR-663a.

In summary, ZNF224 containing KRAB domain functions as an oncogene in breast cancer by regulating p21 and p53 via micro-RNA. It is not surprising any more that ZNF224 functions as a tumor suppressor or oncogene in cancer because there is a possibility that the function of ZNF224 depends on co-activator or repressor bound to KRAB domain of ZNF224. Therefore, it is postulated that there is a co-activator bound to KRAB domain of ZNF224, and it needs their identification and further study to diversify the role of ZNF224.

## MATERIALS AND METHODS

### Cell culture

HEK293 (Human embryonic kidney cell line) and MCF-7 (Human adenoma breast cancer cell line) cells were cultured in Dulbecco's modified Eagle's medium (DMEM, Hyclone, Utah, USA) supplemented with 10% fetal bovine serum (FBS, Hyclone), 1% penicillin/streptomycin (Hyclone, Utah, USA) at 37°C in a humidified 5% CO_2_ incubator. For transfection, plasmid DNA constructs were transfected into cells by polyethylenimine (PEI, Polysciences, Inc., PA, USA) according to the manufacturer's protocol. To obtain overexpressed ZNF224 stable clones, we transfected MCF-7 cells with CMV 3XFlag-ZNF224 G418-selectable plasmid that kindly provided by Dr. Paola Costanzo (University of Naples Federico II, Italy) [10]. Transfected cells were grown and selected in the medium supplemented with 80 μg/ml of G418 (Duchefa, Haarlem, Netherlands). The MCF-7 cells stably overexpressing CMV 3xFLAG-ZNF224 were analyzed by Western blot using anti-FLAG M2 antibody (Sigma Aldrich, MO, USA). To generate ZNF224 knock-down stable cell line, we transfected MCF-7 cells with ZNF224 shRNA plasmid (Santa Cruz Biotechnology, CA, USA). The transfected cells were grown and selected in the medium containing with 10 μg/ml puromycin (Sigma Aldrich, MO, USA). The stable knock-down of ZNF224 was confirmed by Western blot and quantitative RT-PCR.

### RNAi study

MCF-7 cells were transfected with 20 nM ZNF224 si-RNA mixture (#1 5′-CUC AAG ACU UGG UGA UAA ATT-3′, #2 5′-CGA UGU GAU ACG UGU GAU ATT-3′, #3 5′-GGA AAG GGC UAC AAU AGU ATT-3′, Santa Cruz Biotechnology) for 48 h or 72 h, 20 nM miR-663a (5′-AGG CGG GGC GCC GCG GGA CCG C-3′ duplex, Bioneer, Korea) for 48 h or 72 h, and 50 nM miR-663a antagonist (Bioneer, Korea), for 48 h or 72 h using Lipofectamine 2000 (Invitrogen, Paisley, UK) according to the manufacturer's protocol.

### Western blot

For immunoblot, cells were lysed with lysis buffer (50 mM Tris-HCl, pH 7.6, 150 mM NaCl, 1% Triton X-100, 10% glycerol, 1 mM EDTA, 1 mM PMSF, 10 mM NaF, 0.1 mM NaVO_3_). The whole cell lysates (30 μg) were subjected to SDS-PAGE, and transferred to polyvinyldene fluoride membrane (PVDF, Millipore, MA, USA). The membrane was blocked with TBS (20 mM Tris-HCl, pH 7.4, 150 mM NaCl) containing 5% skim milk and 0.2% Tween-20 at room temperature for 1 h, followed by incubation with ZNF224 specific antibody (1:1000, Abcam, Cambridge, UK), M2 anti-FLAG (1:3,000, Sigma Aldrich, MO, USA), anti-p21 (1:1,000, Santa Cruz Biotechnology, CA, USA), anti-p53 (1:1,000, Santa Cruz Biotechnology, CA, USA), anti-PARP1 (1:1,000, Santa Cruz Biotechnology, CA, USA) and anti-alpha-tubulin antibody (1:20,000). The secondary antibodies were anti-mouse IgG HRP (1:25,000, Thermo Scientific, MA, USA) or anti-rabbit IgG HRP (1:30,000, Thermo Scientific, MA, USA). The membrane was developed using enhanced chmiluminescence (ECL) solution (Abclon, Korea).

### Cell growth and drug resistance analysis

For analysis of cell growth and drug resistance, each of stable clones (5×10^4^ cells) was seeded onto 8-E plate (ACEA, CA, USA) and cultured in a humidified 5% CO_2_ incubator. After 10 h, cells were treated with DMSO or 0.1 μM CPT, and further cultured for 24 h. During cell culture, cell growth was monitored in real time using iCelligence system (ACEA, CA, USA).

### Flow cytometry

Apoptosis by CPT was measured by flow cytometry. Cells (2.5×10^6^) were seeded onto 100 mm dish and cultured for 24 h. Then, DMSO or CPT (0.1 μM) was added to each group of cells. After 24 h, cells harvested and fixed in cold 70% ethanol for 16 h. After rehydration with 1x cold PBS, cells were treated with RNaseA (50 μg/ml, Roche, Mannheim, Germany) and propidium iodide (100 μg/ml, Sigma Aldrich, MO, USA). The DNA contents were analyzed by flow cytometry (CUBE6, PARTEC, Germany).

### Chromatin immnunoprecipitation assay and sequencing

HEK293 cells were transfected with CMV 3xFLAG-ZNF224 plasmid (6 μg). Chromatin immunoprecipitation was performed as described previously [46]. Briefly, cells were allowed to crosslink with 1% formaldehyde for 10 minutes at room temperature (RT). The cell pellets were incubated in 200 μl of sonication buffer (50 mM HEPES, pH 7.9, 140 mM NaCl, 1 mM EDTA, 0.1% Na-deoxycholate, 0.1% SDS, 1% Triton X-100 and PIC). The cell lysate was sonicated for 70 seconds (10 sec pulse and 60 sec rest), and DNA lengths were sheared to between 200 and 1,000 bp. The mixture of nucleic acid and protein was incubated with M2 anti-FLAG antibody (Sigma Aldrich, MO, USA) or normal mouse IgG antibody (Santa Cruz Biotechnology, CA, USA), followed by immunoprecipitation using Protein A and G agarose bead mixture (Invitrogen, Paisley, UK). The protein–DNA complexes were separated from the beads by elution buffer (50 mM Tris-HCl, pH 8.0, 1 mM EDTA, 1% SDS, 50 mM NaHCO_3_) and heated at 65°C for 5 h with protease K to reverse the formaldehyde crosslink. Finally, the DNA was purified with the PCR Clean-up Kit (Promega, WI, USA). ChIPed DNA was further processed to prepare sequencing library with some modifications [47]. DNA fragments were end-repaired, dA-tailed, and ligated with Genomic DNA Adapters provided by Illumina. Adapter-ligated DNA fragments were purified and amplified with 20 cycles of PCR. Gel extraction was performed to isolate 200-400 bp of PCR products. Libraries were sequenced by Illumina Genome Analyzer IIx. Alignment of sequences reads were performed using Illumina's CASAVA pipeline (v1.8.2). Uniquely aligned tags allowing 2 mismatches were used for the further analyses. Raw sequencing reads were investigated for quality using FASTQC tool. The reads were aligned to the human genome build hg18 (NCBI 36) using CASAVA v1.6.0 with default parameters. Total mapped tag counts for ZNF224 were 4.09 million. The sequencing data was deposited into the Gene Expression Omnibus (GEO) at the National Center for Biotechnical Information (NCBI) (GSE73947). To identify peaks enriched with FLAG-ZNF224, MACS (Model-based Analysis for ChIP-Seq) ver. 1.4.0 was used with the following options; --tsize=36 --pvalue=1e-4 --mfold=30 --bw=100. Alternatively, HOMER (Hypergeometric Optimization of Motif EnRichment) ver. 4.1 was used with the following options; -fragLength 200 -gsize 2700000000 -center. Annotating peaks in genome was performed with HOMER. A histogram was created by calculating read densities around individual transcription start site (TSS) in each 50 bps. DAVID was used to investigate Gene Ontology terms enriched with genes containing any peaks within 1 kb from their TSSs. Discovery of enriched motifs in peaks were also performed with HOMER.

### ZNF224 purification from HEK293

HEK293 cells were transfected with CMV 3xFLAG-ZNF224 plasmid. After 36 h, protein extracts were prepared with lysis buffer (50 mM Tris-HCl, pH 7.6, 150 mM NaCl, 1% Triton X-100, 10% glycerol, 1 mM EDTA, 1 mM PMSF, 10 mM NaF, 0.1 mM NaVO_3_). The protein extracts (10 mg) were incubated with anti-FLAG M2 magnetic bead (Sigma Aldrich, MO, USA) at 4°C. The precipitated bead-protein complex was washed 1x cold PBS containing 1 mM PMSF, and eluted with elution buffer (0.1 M glycin, pH 2.5). After purification, FLAG-ZNF224 proteins were dialyzed against 1x PBS, and stored at −70°C (Supplementary Figure 7).

### ELISA

To determine DNA consensus sequence recognized by ZNF224, streptavidin coated plate (Thermo Scientific, MA, USA) was washed with 200 μl of wash buffer (25 mM Tris-HCl, pH 7.6, 150 mM NaCl, 0.1% BSA, 0.05% Tween-20), and then 100 μl of the 5′-biotinylated oligo DNA (5 μM) was added to each well. 50 ng of purified FLAG-ZNF224 proteins in 1X PBS containing 20% glycerol was added to each well and incubated for 2 h at RT, and plate was washed three times with wash buffer. Anti-FLAG antibody (1 μg/ml) diluted in wash buffer was added to each well (100 μl/well), and further incubated for 2 h at RT. After washing, the horseradish peroxidatse (HRP)-conjugated goat anti-mouse IgG (1:5,000) diluted in 25 mM Tris-HCl, pH 7.6, 150 mM NaCl, 0.5% BSA, 0.05% Tween-20 was added to each well and further incubated for 30 min. After washing the plate with washing buffer, 3, 3′, 5, 5′ - tetramethylbenzidine solution was added for 15 min. The signal was read at 450 nm (BMG Labtech, Ortenberg, Germay).

### Luciferase activity assay

MCF-7 cells (2.5×10^5^) were seeded onto 12-well plates and transfected with the pGL3-basic luciferase reporter vectors (200 ng) containing triple repeat of CAGCTTC sequence with *Renilla* vector (100 ng) in the presence or absence of FLAG-ZNF224 (200 ng) and cultured for 24 h. To investigate the effect of ZNF224 and miR-663a, MCF-7 cells were transfected with psiCHECK™ vectors (200 ng) containing p21 3′; UTR or p53 3′; UTR with *Renilla* vector (200 ng) in the presence of FLAG-ZNF224 (200 ng) or miR-663a (20 nM) for 24 h. Then cells were treated with miR-663a inhibitor (50 nM, Bioneer, Korea) for 48 h. Luciferase activity was measured with the Dual-Luciferase Reporter Assay System (Promega, WI, USA) and quantified using GloMax^®^ (Promega, WI, USA) according to the manufacturer's instructions.

### Surface plasmon resonance

Surface plasmon resonance (SPR) analysis was performed using a SR7500DC (Reichert, NY, USA). FLAG-ZNF224 proteins (50 μg) were immobilized to CMDH chips (Reichert, NY, USA) according to manufacturer's protocols. Oligo DNA (0, 0.625 μM, 1.25 μM, 2.5 μM, 5.0 μM, and 10 μM) was allowed to pass as an analyte in 1x PBS. K_D_ value was evaluated using the Scrubber2 software.

### Quantitative RT-PCR

To analyze gene expression, total RNA was extracted using TRIzol reagent (Invitrogen, Paisley, UK) according to the manufacturer's protocol. cDNA was prepared by reverse transcription with 500 ng of total RNA, and each gene transcript was amplified by PCR with their specific primers (Supplementary Table 2). Quantitative RT-PCR was performed with Power SYBR Green PCR Master Mix^®^ (ABI, CA, USA) and StepOne 48 well real time PCR system (ABI, CA, USA). To further confirm ChIP sequencing results, Re-ChIP was performed and ChIPed DNA was amplified by PCR with their specific primers (Supplementary Table 3). Each Ct value was normalized to the input samples, ΔCt[Ct(IP) – Ct(Input)]. ChIP signals were calculated as fold value of the input 2^−[ΔΔCt]^. Each ChIP assay was repeated at least three times.

### Immunohistochemical staining

Written informed consent was obtained from breast cancer patients, and this study was approved by Institutional Review Board of Yonsei University Severance Hospital (4-2015-0168). Specimens were ductal carcinoma between stage 1 and 2. Immunohistochemical staining was performed as described previously [48]. Sections (5 μm) were stained for 1 h at RT with anti-p53 and anti-ZNF224 antibodies diluted with 1/50. Staining was detected with the Ultra View universal DAB detection kit (Ventana, AZ, USA) according to the manufacturer's instrutions. The nuclei were counterstained with Mayer's haematoxylin (Dako, Glostrup, Denmark)

### Statistics

The results are expressed mean ± S.D. or mean ± SEM. The student's t test was performed to determine the statistical significance of group all the experiments performed. *p*<0.05 was considered statistically significant.

## SUPPLEMENTARY FIGURES AND TABLES




