# Supplementary material for: ZNF224, Krüppel like zinc finger protein, induces cell growth and apoptosis-resistance by down-regulation of p21 and p53 via miR-663a
Source: Oncotarget. 2016 Apr 20;7(21):31177–90. doi: 10.18632/oncotarget.8870 (PMC5058748; doi:10.18632/oncotarget.8870)
Supplement: Supplementary file 1 [file oncotarget-07-31177-s001.pdf]

SUPPLEMENTARY FIGURES AND TABLES

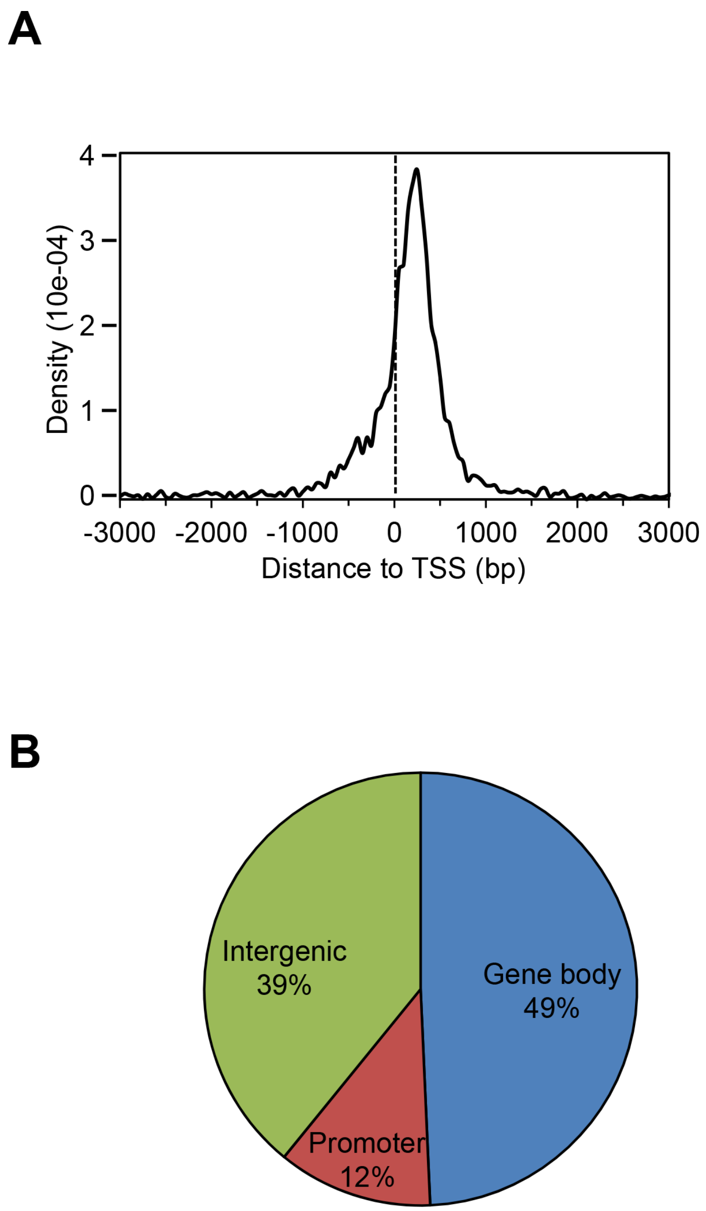

Supplementary Figure S1: Distribution of ZNF224 enriched regions within the ChIP-Seq library. A. Density plot depicting the distribution of enriched regions that are ±1.0 kb from the transcription start site (TSS). **B.** The binding distribution of ZNF224 was calculated in the promoter (±1 kb regions from TSS), gene body, and intergenic regions.

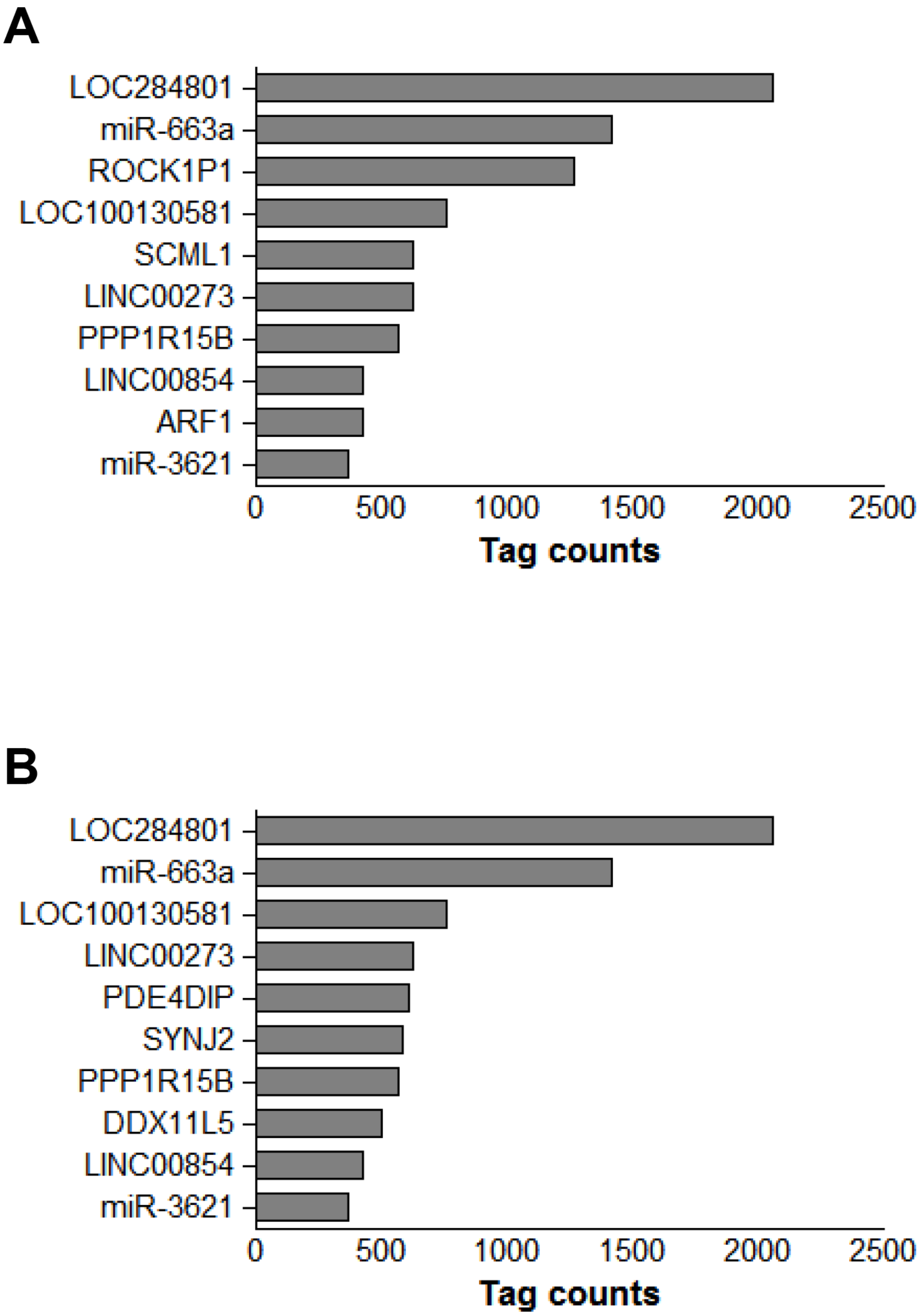

Supplementary Figure S2: List of Top 10 hits from ChIP sequencing. A. Top 10 tags containing 5′-CAGC-3′ among promoter enriched tags of ChIP sequencing. **B.** Top 10 candidates from promoter enriched tags of ChIP sequencing.

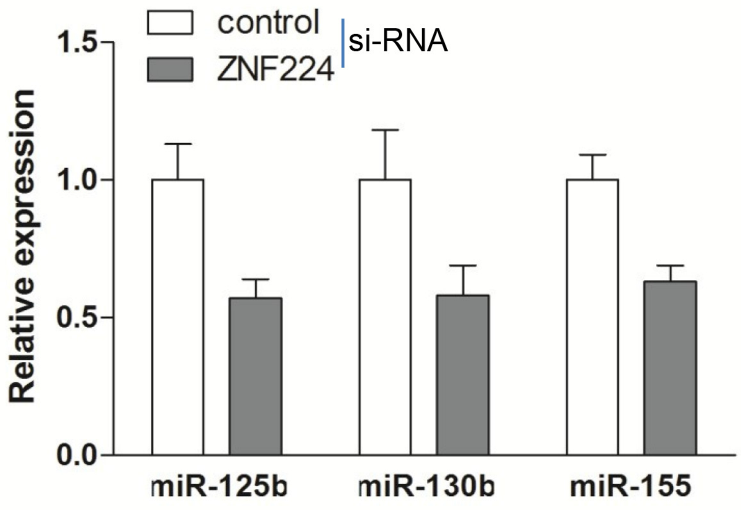

Supplementary Figure S3: Expression of micro-RNA in microarray. Control or ZNF224 siRNA (20 nM) was transfected into MCF-7 cells for 48 h, and the expression levels of miR-125b, miR-130b, and miR-155 were examined.

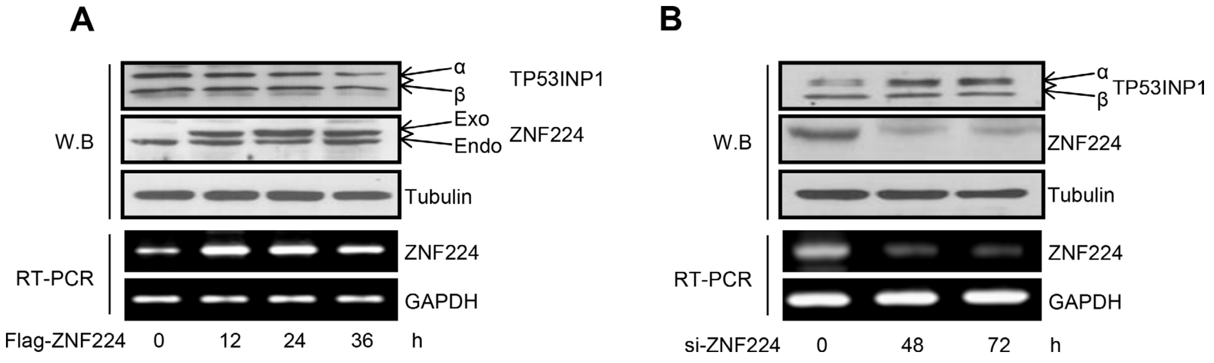

Supplementary Figure S4: ZNF24 regulates the expression of TP53INP1. A-B. FLAG-ZNF224 (2 µg) or ZNF224 si-RNA (20 nM) was transfected into MCF-7 cells for the indicated time. Total RNA and protein extracts were subjected to RT-PCR and immunoblot, respectively. GAPDH and tubulin were used as loading control in RT-PCR and immunoblot, respectively. Data are from at least three independent experiments (n=3).

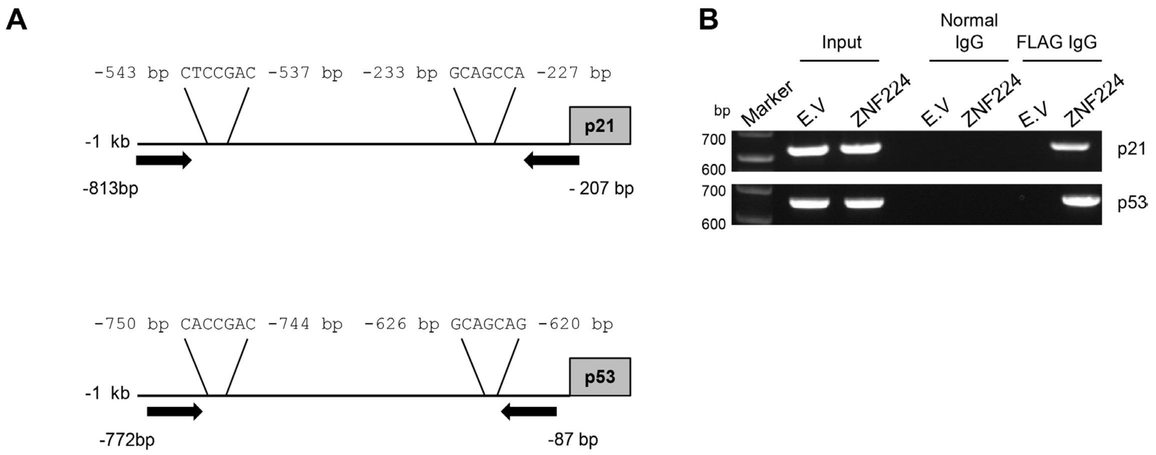

Supplementary Figure S5: ChIP analysis using FLAG-ZNF224. A. Putative binding sequence of ZNF224 in the promoter region of p21 and p53. Arrow indicates primer binding site for PCR. **B.** FLAG-ZNF224 (5 µg) was transfected into HEK293 cells for 24 h, and ChIP assay was performed as described in the Methods section. PCR was performed using p21 and p53 specific primers.

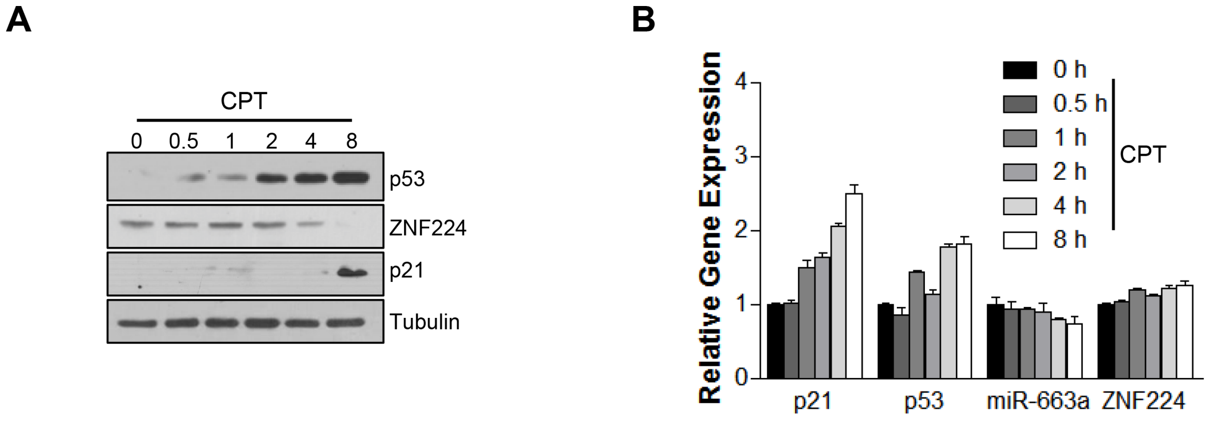

Supplementary Figure S6: The expression levels of p53 and p21 after CTP treatment. MCF-7 cells were treated with CPT (0.1 µM) as indicated, and proteins or total RNAs were harvested for immunoblot **A.** or qRT-PCR **B.** Tubulin, U6 and GAPDH were used as loading controls for immunoblot, miR663a and mRNA analyses, respectively.

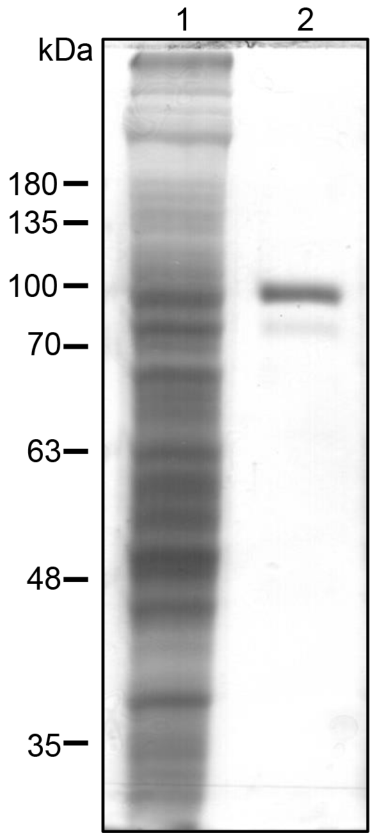

Supplementary Figure S7: Purification of FLAG-ZNF224. HEK293 cells were transfected with FLAG-ZNF224 plasmid for 36 h. After cell lysis as described in the Methods section, FLAG-ZNF224 was purified using the anti-FLAG M2 magnetic bead at 4°C (lane 1, whole cell lysate; lane 2, purified FLAG-ZNF224).

Supplementary Table S1: Genes that contain a ZNF224 enriched region within 1 kb of the transcription start site, within 1 kb of the transcription stop site, and/or within the transcribed sequences

See Supplementary File 1

Supplementary Table S2: Primers used for RT-PCR

Primers	Sequences
GAPDH	F: 5′-CGAGATCCCTCCAAAATCAA-3′<br>R: 5′-TGTGGTCATGAGTCCTTCCA-3′
GAPDH	F: 5′-GGGTGTGAACCATGAGAAGTATG-3′<br>R: 5′-GTCCTTCCACGATACCAAAGTTG-3′
ZNF224	F: 5′-CAGAGAGTCCACATGGGAGAG-3′<br>R: 5′-CCCGTGTGGACCATATGATGC-3′
ZNF224	F: 5′-CACCAGAAGGTCCACACAGG-3′<br>R: 5′-CCCAGCCAAAACTCTTCCCA-3′
P21	F: 5′-GCAGACCAGCATGACAGATTT-3′<br>R: 5′-GGATTAGGGCTTCCTCTTGGA-3′
P53	F: 5′-CCCAAGCAATGGATGATTTGA-3′<br>R: 5′-GGCATTCTGGGAGCTTCATCT-3′
U6	F: 5′-GCTTCGGCAGCACATATACTAAA-3′<br>R: 5′-CGCTTCACGAATTTGCGTGTCAT-3′
miR-663a	F: 5′-AGCCGCGTCCCAACCCGCTAG-3′<br>R: 5′-CTCGCTTGCAGAGGAACCCTC-3′

Supplementary Table S3: Primers used for ChIP qPCR

Primers	Sequences
LOC284801	F: 5′-AGATTTTCTGCCTTGGCACC-3′<br>R: 5′-TGGACTCTCTCAGGTTGAACG-3′
miR-663a	F: 5′-GCCGCGTCCCAACCCGCTAG-3′<br>R: 5′-ACTCGCTTGCAGAGGAACCC-3′
ROCK1P1	F: 5′-GCACTGGAAAACCGATCGTC-3′<br>R: 5′-TTCATCAGTGCGGCTTTCAA-3′
SCML1	F: 5′-CCACAGAGGTAGGATGAGCC-3′<br>R: 5′-ACGGAGAACTTCCCTGTATGG-3′
PPP1R15B	F: 5′-AGGGAGCACATTTACAGGATGG-3′<br>R: 5′-CAACCGACATTGCTGTTGCT-3′
ZDHHC14	F: 5′-GTAAACGTCCGTGGGGAGAA-3′<br>R: 5′-TCAGGGTCCCCTCCGGCCCCG-3′
miR-3621	F: 5′-AGCGAGAGTGCCGAGCAGC-3′<br>R: 5′-CTGCTGTTGCTGCTGCTGTCG-3′
miR-663a	F: 5′-CTTCCGGCGTCCCAGG-3′<br>R: 5′-CGGGCCACCAGGAAAACA-3′
P21	F: 5′-CAGGCTGGTCTCAAAACTCCT-3′<br>R: 5′-GATGTGAGGAAGGCTCAGTGG-3′
P53	F: 5′-CATCAAGCCCTAGGGCTCCTC-3′<br>R: 5′-ATGGAGTTGGGGAGGAGGGTA-3′
